# Supplementary material for: Association of Plasma Vitamins and Carotenoids, DNA Methylation of LCAT, and Risk of Age-Related Macular Degeneration
Source: Nutrients. 2023 Jun 30;15(13):2985. doi: 10.3390/nu15132985 (PMC10347047; doi:10.3390/nu15132985)
Supplement: Supplementary file 1 [file nutrients-15-02985-s001.zip › nutrients-2417112-supplementary.pdf]

## Methods

A microarray data profile (GSE29801) was downloaded from the Gene Expression Omnibus (GEO) database (http://www.ncbi.nlm.nih.gov/geo/) to screen LCAT mRNA expression in AMD patients. Details of the data profile have been described elsewhere [1]. Briefly, retinal samples were isolated from human donor eyes obtained from the University of Iowa (GSH) and the Lions Eye Bank of Oregon. Medical and ophthalmic histories, a family questionnaire, blood, and sera were obtained from the majority of donors. Global transcriptomic profiling was conducted with the Agilent Whole Human Genome 4 × 44 K in situ oligonucleotide array platform (G4112F, Agilent Technologies, Inc., Santa Clara, CA, USA) using manufacturer instructions. The analysis involved 118 samples from the macular or extramacular region of human donor retina. Two-color universal reference design was utilized to investigate AMD, possible preclinical AMD, and those without reported ocular disease.

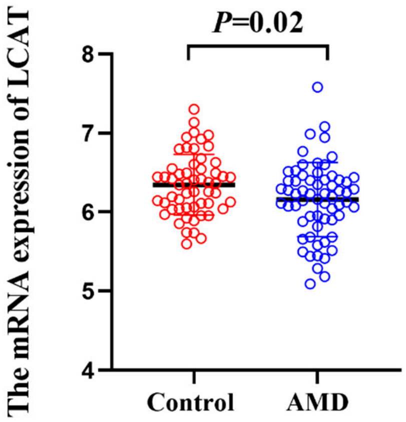


Supplementary Figure S1. The mRNA expression of LCAT is decreased in the retinas of AMD patients. AMD, age-related macular degeneration; LCAT, lecithin-cholesterol aceyltransferase; AMD patients (n=63) and controls (n=55).

**References**

[1] Newman, A.M.; Gallo, N.B.; Hancox, L.S.; Miller, N.J.; Radeke, C.M.; Maloney, M.A.; Cooper, J.B.; Hageman, G.S.; Anderson, D.H.; Johnson, L.V.; Radeke, M.J. Systems-level analysis of age-related macular degeneration reveals global biomarkers and phenotype-specific functional networks. Genome Med. 2012, 4, 16.
